# A comparison of the Fujita classification of awake and drug-induced sleep endoscopy patients

**DOI:** 10.5935/1808-8694.20130017

**Published:** 2015-10-14

**Authors:** Fabio Augusto Winckler Rabelo, Daniel Salgado Küpper, Heidi Haueisen Sander, Vanier dos Santos Júnior, Eric Thuler, Regina Maria França Fernandes, Fabiana Cardoso Pereira Valera

**Affiliations:** PhD in ENT by FMRP-USP (MD, ENT); MSc in ENT by FMRP-USP (Assistant Physician in the ENT Service of HC-FMRP-USP); PhD in Neurology by FMRP-USP (Assistant Physician in the Neurology Service of HC-FMRP-USP); MD, ENT (MD, ENT); PhD in Neurology by FMRP-USP (Professor of Neurology at FMRP - USP); Post-doctoral degree holder (Professor at FMRP - USP). Medical School of Ribeirão Preto - University of São Paulo

**Keywords:** apnea, endoscopy, propofol, sleep apnea, obstructive, sleep apnea syndromes

## Abstract

Only a few studies have compared the outcomes of patients kept awake during endoscopic examination and subjects submitted to drug-induced sleep endoscopy.

**Objective:**

This study aimed to compare the endoscopic findings of patients submitted to outpatient endoscopy and endoscopic examination with sedation by propofol based on the Fujita Classification.

**Method:**

This cross-sectional cohort study enrolled 34 patients. The subjects underwent ENT examination, nasal endoscopy with Müller's maneuver, and drug-induced sleep endoscopy with propofol. The Fujita Classification was used to compare the two modes of endoscopic examination. The examinations were correlated to patient clinical data such as BMI, age, and OSAS severity.

**Results:**

There was no agreement between the two modes of endoscopic examination, whether for the group in general or for the analyzed subgroups.

**Conclusion:**

There was no agreement between the endoscopic findings of endoscopic examinations done with the patient awake or in drug-induced sleep.

## INTRODUCTION

The studies performed on obstructive sleep apnea syndrome (OSAS) have enhanced the understanding of symptoms, facilitated diagnosis, and demonstrated the effectiveness of positive airway pressure therapies^1^. Nonetheless, the difficulties inherent to complying with this mode of treatment have called the attention to other therapeutic possibilities - surgery in particular^1^.

The vast array of approaches and the scarce controlled randomized trials hinder the demonstration of surgery effectiveness. Reviews and meta-analyses aimed at assessing OSAS surgery have presented inconsistent results: Sher et al.^2^ and Sundaram et al.^3^ published meta-analyses on OSAS therapies and found inconsistent results for surgical treatments. Yet, the authors reported that the failure to observe all obstructed sites in the pharynx was the main reason for surgery unsatisfactory results, and that the determination of the site of obstruction should be the main focus of OSAS studies, given its strong correlation with treatment success. In 2010, the American Academy of Sleep Medicine published a review^4^ on various surgical treatments for OSAS and the authors concluded that future studies should focus on the standardization of preoperative evaluation and better patient selection.

In these three important reviews, the authors inferred that the main cause for surgery failure is inaccurate identification of the site of obstruction in the upper airway and, consequently, poor patient selection. The complexity of the upper airways and the multifactorial character of sleep apnea explain the difficulties related to this assessment. The following tests are currently available to aid in patient evaluation: cephalometric measurements, computerized tomography, magnetic resonance imaging, airway mano-metry, fiberoptic laryngoscopy using Müller's maneuver, and drug-induced sleep endoscopy (DISE).

DISE with propofol has been increasingly used, and is currently considered as the endoscopic examination mode that more closely resembles natural sleep and allows for better location of the site of obstruction in the upper airways. Recent studies have ranked highly the effectiveness of this examination in terms of accurately locating sites of obstruction^5^^,^^6^, outcome reproducibility^7^^,^^8^, and patient outcome^9^^,^^10^. Our group recently published a paper^11^ on the polysomnographic alterations introduced by propofol-induced sleep, adding to the reliability of this mode of examination.

However, only a few studies have compared the outcomes of patients kept awake during endoscopic examination and subjects submitted to drug-induced sleep endoscopy. This study is essential in the assessment of whether endoscopic examination under sedation is required.

This study aimed to compare the endoscopic findings of patients submitted to outpatient endoscopy and endoscopic examination with sedation by propofol based on the Fujita Classification.

## METHOD

This is a multicentric study, with the following participating hospitals: University Hospital of the Medical School of Ribeirão Preto - University of São Paulo and the Samaritan Hospital between July of 2006 and January of 2010. All the enrolled patients were educated as to the nature of the study and signed an Informed Consent Form. The research protocol was assessed and approved by the hospital's Ethics Committee in Research with Humans (# 5620/2006).

The sample was made up of patients with a history of snoring and diurnal hypersomnia previously submitted to diagnostic nocturnal polysomnography at the hospital's Sleep Lab using a digital polygraph (Bio- Logic^®^) equipped with analytical software program Sleepscan Vision Analysis version 2.03.05. The following were recorded: electroencephalogram (F3-M2, F4-M1, C3-M2, C4-M1, O1-M2, O2-M1 as per the 10-20 International System), bilateral electroocu-lograms (E1-M2, E2-M1), electrocardiogram (modified V2), surface EMG of the mental and submental muscle, bilateral EMG of the anterior tibial muscle, synchronized digital video (infrared camera - Elbex Inc^TM^), and body position (Netlink body sensor position^TM^). Breathing was monitored as follows: a pressure transducer cannula recorded the flow of air through the nose (Ac Sleep 119, Biolink Medical Br^®^) in combination with a nasal and oral thermal air flow sensor (Pro-Tech thermal air flow sensor^TM^); respiratory inductance plethysmography belts were used to measure respiratory effort (Pro-Tech zRIP respiratory inductance plethysmography^TM^); an oximeter (Netlink Head Box^TM^) was used to assess blood oxygen saturation (O_2_ Sat) and a laryngeal microphone to record respiratory noises. All technical parameters were in accordance with the 2007 Manual of the AASM.

The study enrolled patients with sleep respiratory disorders willing to undergo the tests described below. Thirty-four males (73%) and 12 females (27%) with a mean age of 41.35 ± 7.96 years and a mean BMI of 26.82 ± 3.62 were included. Based on polysomnographic testing, eight patients (17.4%) snored but did not have apnea, 19 (41.3%) had mild OSAS, 10 (21.7%) had moderate OSAS, and nine (19.6%) had severe OSAS.

The exclusion criteria were: age under 18 and above 60 years and patients with cardiorespiratory comorbidities that increased the risk of sedation: previous AMI, congestive heart failure (CHF), severe chronic obstructive pulmonary disease (COPD), etc.

The Fujita Classification was described in 1987 by Fujita and Simmons, and has since been widely utilized in the topographic description of upper airway obstruction. Type I includes isolated oropharyngeal obstruction (and the palatal region); type II features obstruction of the oropharynx and hypopharynx; and type III describes isolated hypopharynx obstruction.

As a routine, during admission patients are submitted to physical and ENT examination, undergo awake fiberoptic laryngoscopy, and are assigned Mallampati and Friedman scores. Radiological examination data (cephalometric measurements, CT and MRI scans) were compiled from the patients' charts. Patients were kept in a seated position during fiberoptic laryngoscopy; the fiberscope was inserted in the nasal cavity after the application of topical vasoconstrictors. Two regions were assessed: na-sopharynx/oropharynx and hypopharynx, at rest and with the patient in Müller's maneuver. At this time, the patients were staged based on the Fujita Classification^12^.

Anesthesia induction with propofol during DISE was carried out by an anesthesiologist. Cardiorespiratory parameters were monitored throughout the examination, and materials for intubation, airway access, and emergency medication were available in the operating room.

Propofol was intravenously administered with the aid of a target controlled infusion pump (Diprifusor^®^ TCI, AstraZeneca) as described in the literature^11^. The estimated mean concentration of propofol on the effector site was 2.34 ± 0.6 mcg/ml.

All DISE procedures were carried out by the same ENT, who used 3.4 mm fiberscopes made by Olympus^®^ or Scad^®^. The following parameters were described for each individual during the examination: velopharyngeal vibration, anteroposterior narrowing of the velopharyngeal lumen, circumferential narrowing of the velopharyngeal lumen, laterolateral narrowing of the oropharyngeal lumen, narrowing of the hypopharynx on the base of the tongue, laterolateral narrowing of the hypopharynx, and narrowing of the hypopharynx on the epiglottis. The patients were then staged based on the Fujita Classification. Obstructions greater than 50% of each level were considered positive.

All the tests described above (polysomnography, awake endoscopic examination, and DISE) were carried out within 90 days.

Weighted Kappa was used to compare the Fujita Classification subject scores on awake endoscopy and DISE and determine the level of agreement between the scores in both circumstances. The impact parameters such as BMI, age, and OSAS severity had upon the level of agreement between the examinations was assessed through ANOVA, Fisher's exact test, and weighted Kappa.

## RESULTS

Findings on velopharyngeal vibration and/or upper airway narrowing during DISE are described on Table 1. Velopharyngeal involvement was seen in 78.26% of the cases, oropharyngeal narrowing in 34.78% of the subjects, and hypopharyngeal narrowing in 54.34% of the subjects. One-level obstruction were seen in 47.83% of the patients, whereas 52.17% of them had multiple levels involved (Table 1).

**Table 1:** Obstruction sites locations in the upper airway see in endoscopy upon induced sleep.

Palate-pharynx	78.26%
Vibration on the palate-pharynx only	15.22%
Anteroposterior palate-pharynx narrowing	26.09%
Circumferential palate-pharynx narrowing	26.09%
End-to-end oropharynx (tonsils)	34.78%
Hypopharynx - tongue-base	41.30%
Hypopharynx - end-to-end	13.04%
Hypopharynx - epiglotis	15.22%
Single level	47.83%
Multiple level	52.17%

The Fujita Classification, based on clinical assessment and DISE, is shown on Table 2. Agreement in the given classifications was observed only in 14 individuals (30.43%).

**Table 2:** Fujita Classification based on outpatient endoscopic examination with the patients awake and drug-induced sleep endoscopy (DISE).

Fujita Classification						
Patient	Gender	Age	OSAS	BMI	Awake endoscopy	DISE
1	Female	39	Mild	24.60	2	3
2	Female	55	Moderate	22.90	2	1
3	Male	33	Mild	25.60	2	3
4	Male	47	Severe	25.30	1	2
5	Male	43	Mild	20.20	2	2
6	Male	51	Moderate	22.10	2	1
7	Male	27	Moderate	27.08	1	1
8	Male	24	Mild	23.87	2	1
9	Female	47	Mild	27.80	1	1
10	Male	46	Moderate	29.80	3	2
11	Male	48	Mild	30.00	3	2
12	Female	53	Moderate	29.10	1	2
13	Female	53	Mild	26.50	2	3
14	Male	28	PS	26.70	2	3
15	Male	44	Moderate	23.90	3	1
16	Male	43	PS	19.90	1	1
17	Male	39	Mild	23.20	1	3
18	Male	50	Mild	28.60	2	3
19	Female	44	PS	27.20	1	1
20	Male	39	Moderate	29.20	1	1
21	Male	43	Mild	28.30	1	3
22	Male	46	Severe	28.40	3	2
23	Male	26	Severe	26.40	2	1
24	Male	26	Moderate	25.50	1	2
25	Female	33	Mild	27.25	1	1
26	Female	43	Mild	22.99	1	2
27	Male	35	Mild	32.65	3	1
28	Male	42	Mild	23.51	1	2
29	Male	42	Mild	28.34	3	3
30	Female	55	Moderate	29.45	3	3
31	Female	53	PS	24.94	1	3
32	Male	38	Mild	27.13	1	2
33	Male	47	Mild	25.03	3	1
34	Male	40	Severe	26.06	3	2
35	Male	33	Mild	25.61	3	1
36	Male	45	Severe	29.07	2	2
37	Male	44	PS	25.93	3	2
38	Male	38	PS	20.83	1	3
39	Male	45	Severe	24.16	2	2
40	Male	46	Moderate	27.78	1	2
41	Female	38	PS	32.42	3	1
42	Male	31	Severe	26.51	3	1
43	Fem	30	Severe	36.93	2	2
44	Male	50	Mild	28.40	3	3
45	Male	42	Severe	37.09	1	1
46	Male	36	PS	22.59	2	3
		41.30 ± 8.18			26.67 ± 3.64	

PS: primary snoring; DISE: drug-induced sleep endoscopy; OSAS: obstructive sleep apnea syndrome.

Table 3 shows the statistical analysis used to assess the level of agreement between the classifications given during both endoscopic examinations. A level of −0.03 was verified, i.e., there was no agreement between the two examinations performed on the same patients. When the groups were subdivided for clinical parameters, the Kappa value was still low (0.01 for patients above 45 years of age; −0.01 for patients under 45; −0.25 for patients with a BMI under 25; 0.08 for patients with a BMI above 25; −0.10 for patients with mild OSAS; 0.10 for patients with moderate or severe OSAS). Therefore, clinical parameters had no impact upon the disagreement seen between the two endoscopic examinations.

**Table 3:** Analysis of Fujita Classification agreement between DISE and clinical assessment (physical examination combined with Müller's maneuver). (Weighted Kappa).

	Clinical Examination with Subjects Awake		
DISE	1	2	3
1	7 (38.9)	4 (28.6)	6 (42.9)
2	7 (38.9)	4 (28,6)	5 (35.7)
3	4 (22.2)	6 (42,9)	3 (21.4)

Weighted Kappa: −0.03 95% CI (−0.24; 0.19); DISE: Drug-Induced Sleep Endoscopy.

## DISCUSSION

Consensus states that the better and more accurate the characterization of the site of obstruction in patients with sleep-disordered breathing (SDB), the better the therapy and its results. The reviews cited in this paper have shown that outpatient endoscopy is not enough to properly stratify patients, as indicated by the inconsistent success rates reported for the analyzed surgical procedures^2^. With this in mind, our group carried out a study to assess the success rates of uvulopalatopharyngoplasty (UPPP) based on clinical, outpatient endoscopic, and polysomnographic criteria, only to observe that 28 (44%) of the 64 patients enrolled in the study recovered completely^13^. This finding reinforces the fact that the approach currently in place -based on outpatient endoscopic criteria and the Friedman Classification - does not adequately select the ideal candidates for palatal surgery.

Drug-induced sleep endoscopy (DISE) has been considered by a number of recent studies as the examination that more closely resembles natural sleep, in addition to enabling dynamic, three-dimensional evaluation of the upper airways as the subject snores or suffers from episodes of apnea^7^^,^^8^. According to these authors, the detailed description of the sites of upper airway obstruction allowed by DISE has facilitated the choice of therapy and led to the wider use of this endoscopic examination in OSAS care centers. Two classification models have been proposed to rate DISE findings: the VOTE^8^ and the NOHL^14^ scales. These schemes enable the observation of collapses in traditionally uninvolved structures in outpatient endoscopic examination, as is the case of collapsed epiglottis seen in 15.22% of our cases. Thus, comparisons by the exact site of obstruction between awake endoscopy and DISE are inherently flawed. This is why we opted to use a simpler and more basic classification system to make this comparison based on the topographic level of obstruction (Fujita Classification).

There was no agreement in the Fujita Classification assigned to patients in awake and sleep-induced endoscopy (Kappa = −0.03). This disagreement may have occurred due to the lack of consideration given to specific conditions and characteristics of sleep, neuromuscular alterations, respiratory and postural patterns in deciding upon a therapy.

Our findings are similar to those reported by Campanini et al.^15^. After assessing 250 patients, these authors found similar classifications in only 25% of the cases, with greater disagreements in obstructions of the hypopharynx. The authors also stressed that in 33% of the cases the examination allowed the observation of relevant laryngeal obstructions that were missed in the examination done with the subjects awake. Gregorio et al.^16^ reported that more retrolingual collapses were seen with DISE than with Müller's maneuver in a group of eight patients, and concluded that outpatient assessment alone may lead to underdiagnosis.

Another important finding as we analyze patients with agreeing or disagreeing Fujita Classifications and compare them for BMI, age, or OSAS severity, is that there is no clinical criteria to support the use of DISE. The lack of a correlation or pattern of obstruction reinforces the idea that each patient must be analyzed in an individualized fashion.

The lack of agreement between the examinations proves that there is a marked neuromuscular component in the obstruction of the airways of subjects in their sleep, and that we are probably underestimating these findings when we base ourselves only on the endoscopic findings of awake patients.

More studies are required to confirm the importance of DISE in the assessment of OSAS patients. However, this study has confirmed that the same endoscopic examination, when performed at different times, may yield conflicting results.

## CONCLUSION

The assessment derived from drug-induced sleep endoscopy differs from the evaluation produced from the endoscopic examination of awake subjects.
